# Shaoyao Gancao decoction alleviates functional constipation by inhibiting *Escherichia-Shigella* expansion, modulating gut microbiota, and suppressing dysbiosis-induced endocannabinoid production: evidence from a self-controlled pilot study

**DOI:** 10.3389/fcimb.2025.1705271

**Published:** 2025-12-09

**Authors:** Liying Zhu, Hua Gao, Qing Li, Yang Wang, Jinjun Li, Xiaoqiong Li, Zhihui Huang, Chi Wang, Jinshan Nie

**Affiliations:** 1 State Key Laboratory for Quality and Safety of Agro-Products, Institute of Food Sciences, Zhejiang Academy of Agricultural Sciences, Hangzhou, China; 2 Department of Gastroenterology, The First People’s Hospital of Taicang, Taicang Affiliated Hospital of Soochow University, Taicang, China; 3 Suzhou Chien-shiung Institute of Technology, Taicang, China; 4 Division of Gastroenterology, Sir Run Run Shaw Hospital, School of Medicine, Zhejiang University, Hangzhou, China; 5 Inner Mongolia Institute of Digestive Diseases, The Second Affiliated Hospital of Baotou Medical College, Baotou, China

**Keywords:** Shaoyao Gancao decoction, functional constipation, gut microbiome, *Escherichia-Shigella*, dysbiosis, retrograde endocannabinoid signaling, short-chain fatty acids

## Abstract

**Introduction:**

Shaoyao Gancao Decoction (SGD), a classical traditional Chinese medicine formula, has been clinically reported to improve symptoms of functional constipation (FC), although its underlying mechanisms remain unclear. This study aimed to explore the clinical efficacy and gut microbiota modulation of SGD in patients with FC.

**Methods:**

A self-controlled pilot study was conducted in 20 patients diagnosed with FC according to the Rome III (IV) criteria. Participants received a 3–5 day oral intervention with SGD. Clinical outcomes, including stool frequency, consistency, and ease of defecation, were evaluated using self-reported questionnaires. Fecal samples collected before and after treatment were analyzed for microbial composition (16S rRNA sequencing) and short-chain fatty acids (SCFAs).

**Results:**

Ninety percent of participants reported symptomatic improvement, with 70% achieving increased stool frequency (> 3 times/week). SGD treatment markedly shifted the fecal microbiota from a dysbiotic state dominated by *Proteobacteria, Enterobacteriaceae*, and *Escherichia–Shigella* to a community enriched in *Firmicutes, Veillonella, Roseburia*, and *Ruminococcus*. These microbial changes were accompanied by significant increases in fecal SCFAs and improvements in stool consistency and frequency. Functional prediction analysis revealed that SGD suppressed unsaturated fatty acid and arachidonic acid metabolism, thereby attenuating retrograde endocannabinoid signaling associated with intestinal hypomotility. Feature taxa enriched in responders—such as *Ruminococcus* sp. N15.MGS-57 and *Bacteroides coprophilus*—were linked to enhanced estrogen activity and secondary bile acid metabolism.

**Discussion:**

These findings suggest that SGD alleviates FC by restoring microbial balance, enhancing SCFA production, and suppressing dysbiosis-induced endocannabinoid signaling. As a pilot study, the results are preliminary but provide mechanistic insights that warrant validation in larger, randomized controlled trials.

## Introduction

Functional constipation (FC) is a common digestive disorder without identifiable organic abnormalities. It disproportionately affects women and shows increasing prevalence with age (Suares and Ford, 2011). Beyond compromising quality of life, FC is associated with an elevated risk of comorbidities, particularly cardiovascular and cerebrovascular diseases in elderly individuals (Guo et al., 2020). Despite its high prevalence, current treatment strategies often have limited long-term efficacy and are associated with undesirable side effects (Vriesman et al., 2020). First-line therapies typically include osmotic and stimulant laxatives (Bashir and Sizar, 2024). However, these agents may induce adverse effects such as abdominal discomfort, bloating, diarrhea, and nausea (Lacy et al., 2016), and chronic use—especially of anthraquinone-based stimulant laxatives—can lead to complications such as melanosis coli (Spiessens et al., 1991). Hence, there is a pressing need for safe and effective pharmacological alternatives for managing FC.

Shaoyao Gancao decoction (SGD), a traditional Chinese herbal formula composed of *Paeonia lactiflora* (white peony root) and *Glycyrrhiza uralensis* (licorice), has shown clinical efficacy in relieving constipation symptoms. Historically recorded in the *Shang Han Lun* by Zhang Zhongjing, a foundational clinical work of Traditional Chinese Medicine compiled during the Eastern Han dynasty (c. 200 AD), SGD was initially used to treat gastrocnemius spasms (Qu et al., 2020). Contemporary clinical practice has revealed its pronounced laxative effects, often without inducing diarrhea or abdominal cramping (Zhu et al., 2017; Qu et al., 2020). The major components in SGD are gallic acid, paeoniflorin, and albiforin (Luo et al., 2023). Preclinical studies suggest that total peony glucosides containing 90.42% paeoniflorin can alleviate constipation by modulating neurotransmitter levels—reducing inhibitory mediators such as nitric oxide (NO) and vasoactive intestinal peptide (VIP)—and by increasing the number of interstitial cells of Cajal, which are essential for gut motility (Zhu et al., 2016; Wang et al., 2013). Notably, in our clinical practice, SGD exerts a rapid, sustained, and non-dose-dependent therapeutic effect in patients with refractory constipation, without the adverse outcomes seen with anthraquinone-based agents.

FC is a multifactorial condition involving genetic, behavioral, and physiological factors, as well as alterations in the gut microbiota (Vriesman et al., 2020). Emerging evidence indicates that FC is associated with shifts in microbial composition (Ceresola et al., 2018; Ohkusa et al., 2019). Additionally, microbial metabolites such as short-chain fatty acids (SCFAs) and bile acids are believed to play a role in FC pathophysiology (Dimidi et al., 2017; Zhang et al., 2021; Wang and Yao, 2021). In particular, a reduced abundance of beneficial bacteria, such as *Lactobacillus* and *Bifidobacterium*, and an increased proportion of potentially pathogenic taxa have been observed in patients with FC (Ohkusa et al., 2019). Chinese clinical studies have also linked FC to elevated levels of *Parabacteroides, Bacteroides*, and *Ruminococcus* (Huang et al., 2018; Tian et al., 2021; Fan et al., 2022; Guo et al., 2020; Tian et al., 2020).

Microbial metabolites—particularly SCFAs and bile acids—are central to gut function and motility. SCFAs, including acetate, propionate, and butyrate, are fermentation products that influence intestinal epithelial health, fluid absorption, and colonic transit (Wang and Yao, 2021). However, their precise role in FC remains ambiguous, with some studies reporting reduced SCFA levels—particularly acetate—in patients with slow transit constipation (STC) and irritable bowel syndrome with constipation (IBS-C) (Tian et al., 2021; Mars et al., 2020). Similarly, reduced bile acid levels have been observed in individuals with FC and IBS-C, and interventions targeting bile acid transport have shown therapeutic promise (Abrahamsson et al., 2008; Wong et al., 2011; Mars et al., 2020).

Recent animal studies suggest that SGD may modulate the host immune response and gut microbial composition. SGD has been shown to attenuate inflammation and reverse dysbiosis in ovalbumin-induced asthma models (He et al., 2023), reduce lipopolysaccharide (LPS)–producing *Proteobacteria* in polycystic ovary syndrome (PCOS) rats (Chang et al., 2021), and increase the abundance of probiotic bacteria in liver injury models (Li et al., 2022). These findings suggest that the microbiome may be a key mediator of SGD’s therapeutic effects. However, the microbial mechanisms by which SGD ameliorates functional constipation remain unclear.

In this study, we investigated fecal microbiota and SCFA profiles in FC patients before and after an SGD intervention using 16S rRNA gene sequencing and metabolite analysis. Our findings suggest that SGD alleviates constipation by correcting microbial dysbiosis and suppressing host endocannabinoid signaling pathways, which may be triggered by microbial-derived lipid mediators. These results provide mechanistic insights into the role of the gut microbiome in SGD-mediated improvements in intestinal motility.

## Materials and methods

### Study subjects

Twenty patients diagnosed with FC were recruited between September and November of 2021. The sample size was consistent with prior pilot microbiome studies, in which cohorts of approximately 20 participants are commonly reported (Jose et al., 2022*;*Mejía-Granados et al., 2022). The study was approved by the Ethics Committee of the First People’s Hospital of Taicang (KY-2019-201) and conducted in accordance with the Declaration of Helsinki. Written informed consent was obtained from all participants.

The inclusion criteria were as follows: (1) diagnosis of FC according to the Rome III (IV) criteria, (2) age ≥ 18 years; (3) disease duration > 6 months. The exclusion criteria included: (1) organic intestinal diseases, (2) history of intestinal surgery, (3) constipation caused by the long-term use of psychotropic or other medications, (4) use of probiotics, prebiotics, or synbiotics within the previous month, and (5) use of antibiotics within the last month.

### Study design and sample collection

Participants received a one-week oral intervention of SGD, administered once daily, one hour before bedtime. Each dose consisted of 5 g of raw licorice and 30 g of raw white peony root, prepared as granules (Jiangsu Jiangyin Tianjiang Pharmaceutical Co., Ltd.) and dissolved in 150 mL of warm water.

Self-reported questionnaires were completed before and after the intervention, assessing weekly spontaneous bowel movement frequency, stool consistency (Bristol Stool Scale), ease of defecation, and perceived overall efficacy. The Defecation Smoothness Score (graded A–C) and Efficacy Score (graded A–D) are defined in **Supplementary Table 1**. Responder and non-responder classification was based primarily on the objective criterion of stool frequency: participants with >3 bowel movements per week post-intervention were classified as responders, while those with ≤3 bowel movements per week were classified as non-responders. In addition, participants with both an *Efficacy Score* and *Defecation Smoothness Score* of C were also categorized as non-responders, regardless of stool frequency.

Fecal samples were collected before treatment and again 3–5 days post-treatment. The samples were immediately stored at −80 °C for subsequent microbiological and SCFA analyses.

### SCFA determination

SCFA concentrations were measured via gas chromatography as previously described (Mao et al., 2013). Briefly, 1 mL of a fecal slurry (10% w/v) was acidified with 0.2 mL of 25% (w/v) metaphosphoric acid, followed by centrifugation at 14,000 g for 5 min. The resulting supernatant was analyzed using a Shimadzu GC-2010 Plus (Japan) with a DB-FFAP column (0.32 mm × 30 m × 0.5 μm; Agilent Technologies, USA) and a hydrogen flame ionization detector. Crotonic acid was used as the internal standard. Quantified SCFAs included acetic, propionic, isobutyric, butyric, isovaleric, and valeric acids.

### DNA extraction

Bacterial genomic DNA was extracted from fecal samples using the QIAamp DNA Stool Mini Kit (Qiagen, Germany) following the manufacturer’s instructions. The extracted DNA was stored at −80 °C for sequencing.

### 16S rRNA gene sequencing and bioinformatics analysis

DNA from pre- and *post*-intervention samples *was* analyzed by Novogene Bioinformatics Technology Co., Ltd. (Beijing, China). The V4*–*V5 region of the 16S rRNA gene was amplified using primers 515F (5’-GTGCCAGCMGCCGCGGTAA-3’) and 907R (5’-CCGTCAATTCCTTTGAGTTT-3’). Libraries were prepared using the NEBNext Ultra II DNA Library Prep Kit (Illumina, USA), and library quality was assessed via Qubit. Paired-end sequencing (250 bp) was performed on the Illumina NovaSeq platform.

Data processing—including raw read merging, quality filtering, chimera removal, and sequence assembly—was conducted using QIIME2 (version 2022.2). Amplicon sequence variants (ASVs) were generated using DADA2 or the Deblur plugin and taxonomically classified with the Silva database. Alpha diversity (Chao1, Shannon, and Simpson indices) and beta diversity (Bray–Curtis distances) were calculated in QIIME2. PCoA was visualized using ade4 and ggplot2 in R (v4.0.3).

Statistical analysis of beta diversity (Adonis) was performed using vegan, and LEfSe (LDA Effect Size) analysis was carried out in R. Spearman’s rank correlations were used to evaluate associations between microbial taxa, SCFAs, and clinical outcomes. Functional potential was inferred using PICRUSt2 (v2.3.0) against the KEGG database, and microbial phenotypes were predicted using BugBase (Ward et al., 2017*;*Zhang et al., 2020). The sequencing data are available in the National Microbiology Data Center under the accession number NMDC10018777 (https://nmdc.cn/resource/genomics/sra/detail/NMDC10018777).

### Statistical analysis

All statistical analyses were conducted using GraphPad Prism 9.5.1 (GraphPad Software, Inc., San Diego, CA, USA). Wilcoxon matched-pairs signed-rank tests were used to assess differences before and after the intervention for all patients, including responders and non-responders. Mann–Whitney U tests were used for comparisons between responders and non-responders. A p-value <0.05 was considered statistically significant.

## Results

### Participant characteristics and clinical evaluation

Twenty participants with FC (Rome III (IV) criteria) completed the study. Baseline and post-intervention clinical data are summarized in **Supplementary Table 1**.

### Clinical efficacy of SGD

**Figure 1:**
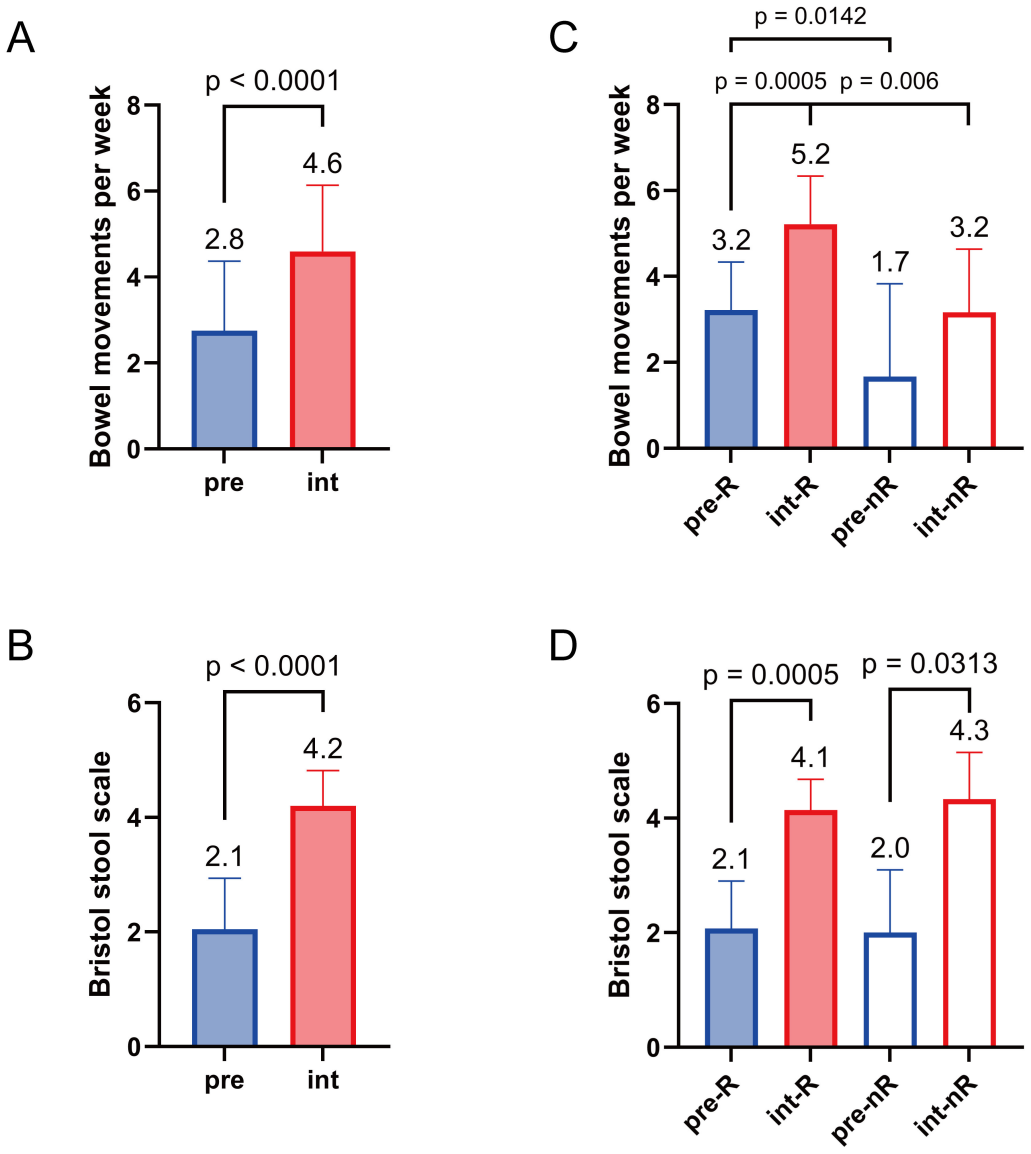
Comparison of outcomes after SGD intervention. Weekly bowel movements were compared before (pre) and after (int) the SGD intervention in all patients **(A)** and between responders (R) and non-responders (nR) **(B)**. Bristol Stool Scale scores were also compared in all patients **(C)** and between responders and non-responders **(D)**. The mean value for each outcome is shown in the bar plot. Wilcoxon matched-pairs signed-rank tests were performed for pre vs. int, preR vs. intR, and prenR vs. intnR; Mann–Whitney tests were used for preR vs. prenR and intR vs. intnR.

Following the SGD intervention, stool frequency and consistency significantly improved (**Figures 1A, B**). The mean number of bowel *movements* and Bristol Stool Scale scores increased significantly. Overall, 90% of participants reported some improvement in global efficacy, and 30% reported significant relief (**Table 1**). Ease of defecation improved in 95% of patients, with 65% showing marked improvement.

**Table 1:** Demographic and clinical characteristics of patients.

Characteristics	Patient	Responders	Non-responders
age*	42.8 ± 14.9	40.5 ± 12.9	48.2 ± 19.1
BMI*	21.8 ± 1.8	21.5 ± 1.8	22.5 ± 1.5
n	20	14	6
Male participants (%)	2 (10%)	1 (7%)	1 (17%)
Female participants (%)	18 (90%)	13 (93%)	5 (83%)
Global efficacy			
markedly improved	6 (30%)	6 (43%)	0
slightly improved	12 (60%)	7 (50%)	5 (83%)
unchanged	2 (10%)	1 (7%)	1 (17%)
worse	0	0	0
Ease of bowel movement			
markedly improved	13 (65%)	12 (86%)	1 (16.7%)
slightly improved	6 (30%)	2 (14%)	4 (66.6%)
unchanged	1 (5%)	0	1 (16.7%)
worse	0	0	0

*The data are shown as the mean ± SD.

According to the predefined criteria (Materials and Methods; **Supplementary Table 1**), 14 participants were classified as responders, defined as having >3 bowel movements per week post-intervention, yielding an overall efficacy rate of 70%. The remaining six participants were classified as non-responders—five with ≤3 bowel movements/week and one with both global efficacy and defecation smoothness scored as C. Due to the limited number of male participants (n = 2, one in each response group), sex-stratified analyses were not performed.

### Comparison between responders and non-responders

Responders exhibited a significant increase in both stool frequency and consistency, whereas non-responders improved only in stool consistency (**Figures 1C, D**). Non-responders consistently exhibited lower stool frequency at baseline and after treatment. Among the responders, 100% reported improved defecation ease, and 93% noted enhanced global efficacy (**Table 1**). In contrast, only 83% of non-responders reported improvements in both measures. These findings indicate that the microbial and metabolic changes observed in responders may be more closely associated with the therapeutic effects of SGD.

### Changes in microbial diversity

**Figure 2:**
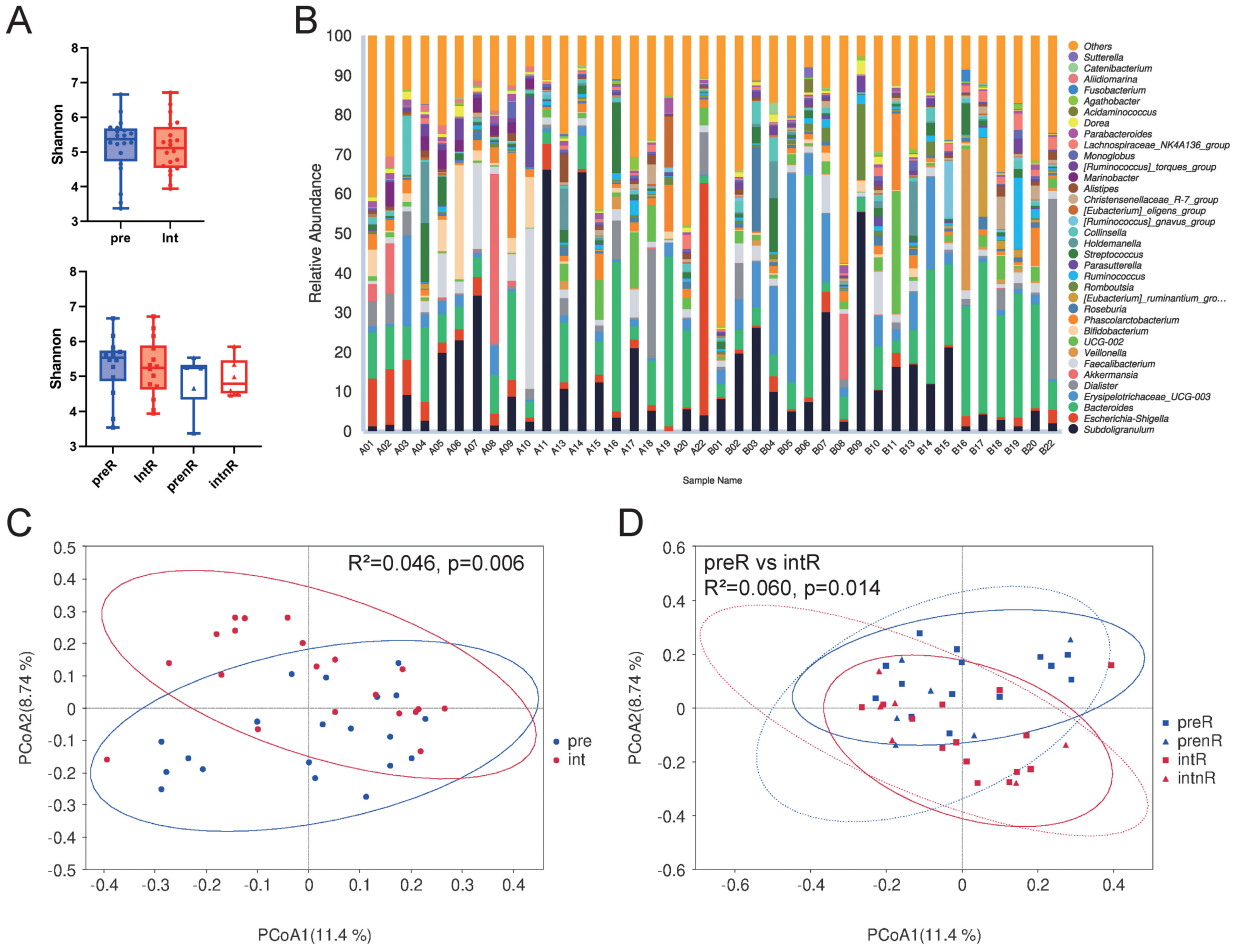
Differences in α-diversity and β-diversity in fecal microbiota before and after SGD intervention. **(A)** Shannon index of all patients before (pre) and after (int) the SGD intervention and among four subgroups: responders before (preR) and after the intervention (intR) and non-responders before (prenR) and after the intervention (intnR). Wilcoxon matched-pairs signed-rank tests were performed for pre vs. int, preR vs. intR, and prenR vs. intnR; Mann–Whitney tests were used for preR vs. prenR and intR vs. intnR. **(B)** Relative abundance of the top 35 genera in each group. **(C)** PCoA of all patients before (blue) and after (red) the intervention using the Bray–Curtis distance matrix. **(D)** PCoA of the four subgroups before (blue) and after (red) the intervention; responders (solid line) and non-responders (dotted line). *P-values <*0.05 were calculated using the Adonis test.

Microbial alpha diversity (**Figure 2A**) did not significantly change following SGD treatment. However, beta diversity (PCoA based on Bray–Curtis distances) showed significant changes in the overall cohort and in responders (**Figures 2C, D**, Adonis test), suggesting an alteration in community composition. The relative abundance at the genus level is shown in **Figure 2B**.

### Microbial composition shifts

**Figure 3:**
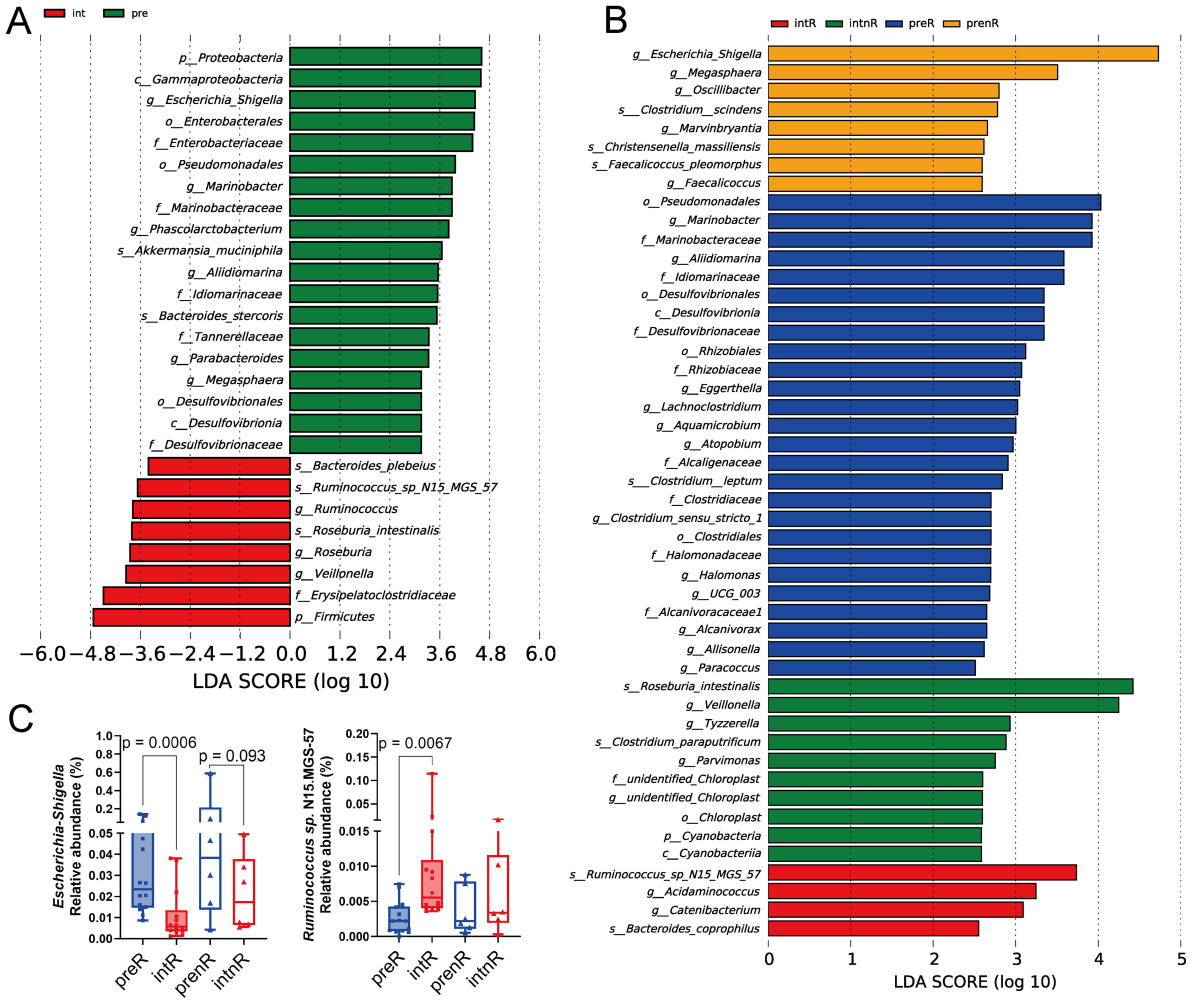
LEfSe of fecal microbiota before and after SGD intervention. **(A)** LEfSe of all patients before (pre) and after (int) the intervention, LDA >3.15. **(B)** LEfSe of the four subgroups: responders before (preR) and after (intR) the intervention and non-responders before (prenR) and after (intnR) the intervention, LDA >2.47. **(C)** Relative abundance of *Escherichia–Shigella* and *Ruminococcus* sp. N15.MGS-57 was compared among subgroups. Wilcoxon matched-pairs signed-rank tests were used; *p <*0.1 for plots shown.

LEfSe analysis identified 24 taxa enriched at baseline and 10 taxa predominating after treatment (**Figure 3A**). Following the SGD intervention, *Firmicutes* replaced *Proteobacteria* as the predominant phylum. At the genus level, *Escherichia*–*Shigella* was dominant at baseline, along with its higher taxa—*Enterobacteriaceae*, *Enterobacteriales*, and *Gammaproteobacteria*. After treatment, genera such as *Veillonella*, *Roseburia*, and *Ruminococcus* became prevalent. Other genera enriched at baseline included *Phascolarctobacterium*, *Parabacteroides*, *Marinobacter*, *Aliidiomarina*, and *Megasphaera*.

Subgroup analysis (**Figure 3B**) further revealed distinct genus-level patterns between responders and non-responders. *Megasphaera* was enriched in non-responders alongside *Escherichia*–*Shigella*, whereas *Marinobacter* and *Aliidiomarina* were characteristic of responders at baseline. Additionally, *Oscillibacter*, *Marvinbryantia*, and *Faecalicoccus* predominated in non-responders, while *Eggerthella*, *Aquamicrobium*, *Atopobium*, *Clostridium sensu stricto 1*, and *Halomonas* were signature genera in responders. These findings suggest that differences in baseline microbial composition may underlie differential treatment responses.

After the SGD intervention, the post-treatment microbiota also differed between subgroups. *Acidaminococcus*, *Catenibacterium*, *Ruminococcus* sp. N15.MGS-57, and *Bacteroides coprophilus* were enriched in responders, whereas *Veillonella*, *Tyzzerella*, *Roseburia intestinalis*, and *Clostridium paraputrificum* predominated in non-responders. Notably, *Ruminococcus* sp. N15.MGS-57 was a characteristic post-treatment species in both responders and the overall cohort, with a significant increase observed only among responders (**Figure 3C**, right). Similarly, *Escherichia*–*Shigella* decreased in both groups, but statistical significance was achieved only in responders (**Figure 3C**, left).

### SCFA profile changes

**Figure 4:**
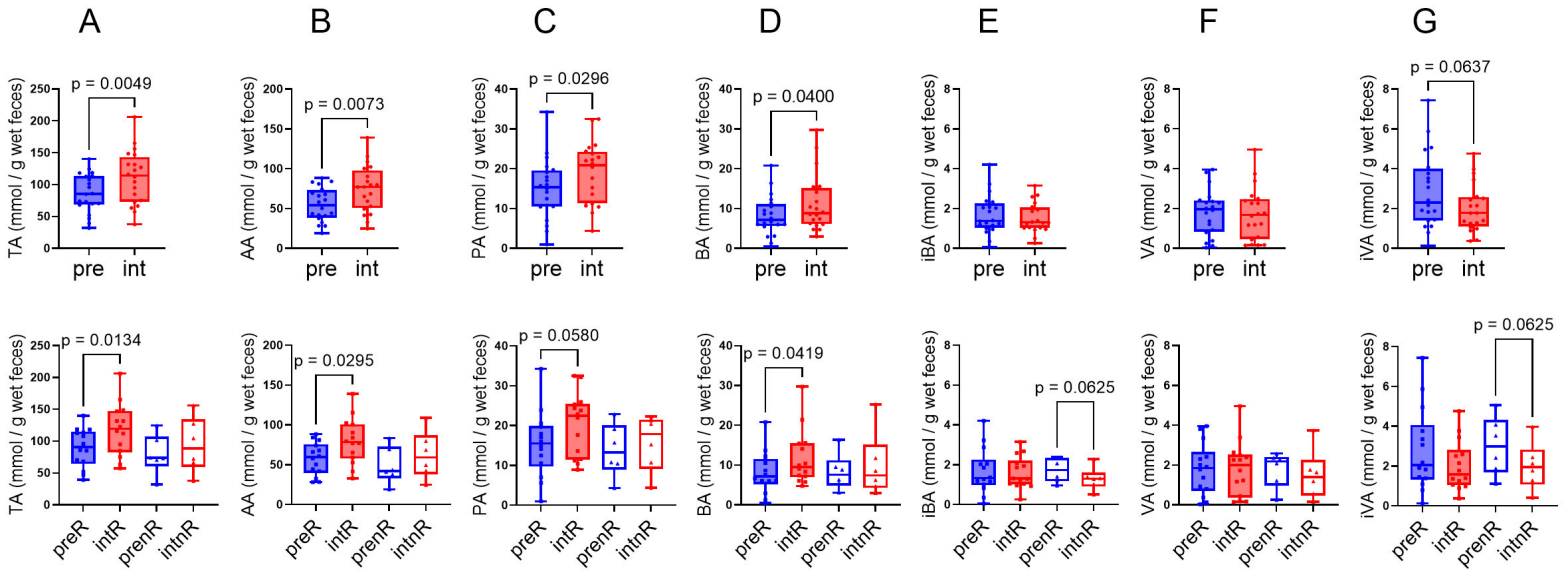
Comparison of SCFAs before and after SGD intervention. SCFAs were determined by GC. **(A)** total SCFAs (TA), the sum of six acids; **(B)** acetic acid (AA); **(C)** propionic acid (PA); **(D)** butyric acid (BA); **(E)** isobutyric acid (iBA); **(F)** valeric acid (VA); **(G)** isovaleric acid (iVA). pre, before the intervention; int, after the intervention; preR, responders before the intervention; prenR, non-responders before the intervention; intR, responders after the intervention; intnR, non-responders after the intervention. *p <*0.1 for plots shown.

SGD significantly increased total SCFA levels, especially acetate, propionate, and butyrate, while branched-chain SCFAs (isobutyrate, valerate, and isovalerate) showed a decreasing trend (**Figure 4**). Subgroup analysis showed significant SCFA increases only among responders. The decline in branched-chain SCFAs was more pronounced in non-responders, suggesting that SGD modulates SCFA metabolism more effectively in responsive individuals.

### Correlation of microbial taxa with SCFAs and clinical outcomes

**Figure 5:**
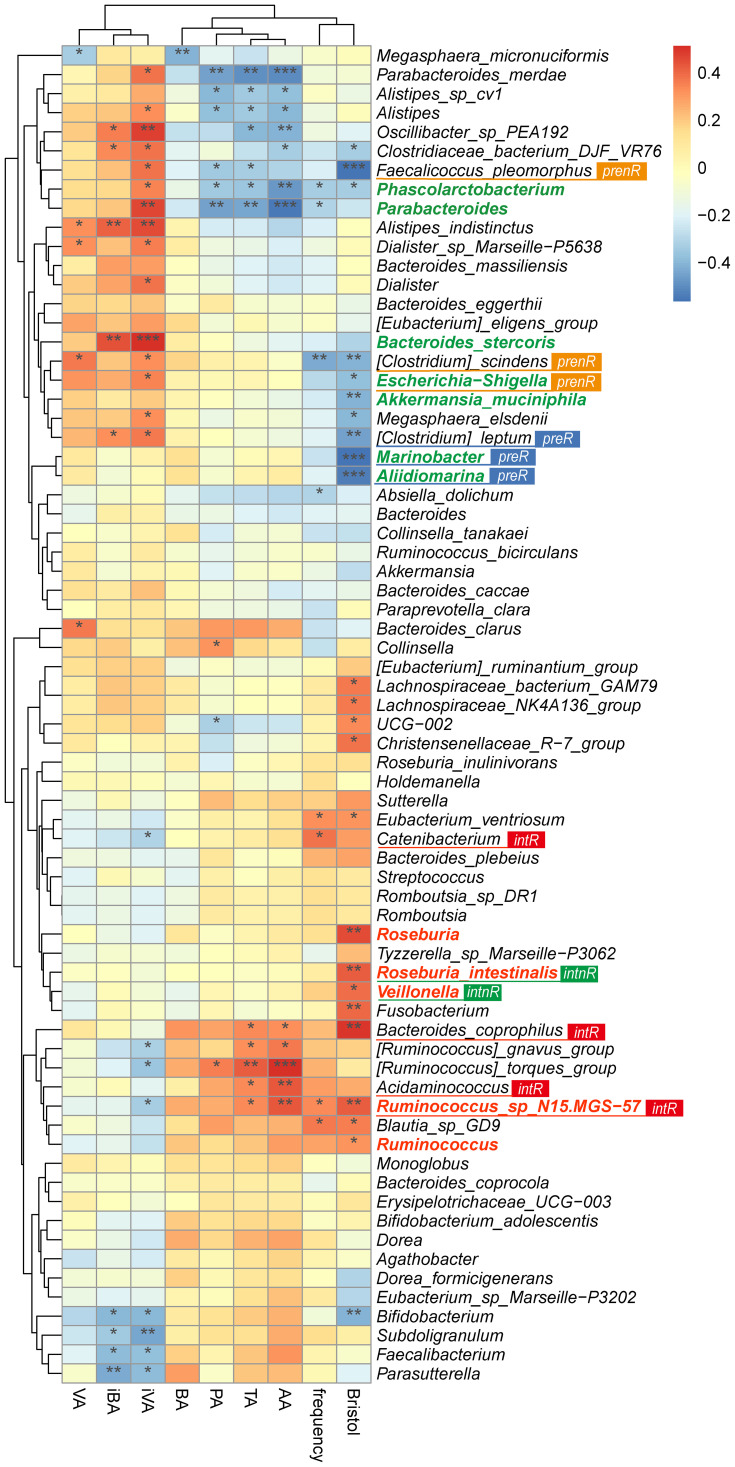
Correlation heatmap of the top 35 genera and species with SCFAs and clinical outcomes. Spearman’s rank correlation coefficient analysis revealed correlations between the top 35 genera and species, clinical outcomes, and SCFAs. Outcomes include weekly bowel movements (frequency) and Bristol Stool Scale score (Bristol). SCFAs include total SCFAs (TA), acetic acid (AA), propionic acid (PA), butyric acid (BA), isobutyric acid (iBA), valeric acid (VA), and isovaleric acid (iVA). Feature taxa identified by LEfSe at baseline and post-intervention are shown in green and red, respectively. Signature taxa in each subgroup are labeled and colored consistently with **Figure 3B**. preR, responders before the intervention; prenR, non-responders before the intervention; intR, responders after the intervention; intnR, non-responders after the intervention. *p <0.05; **p <0.01; ***p <0.001.

Spearman’s correlation analysis (**Figure 5**) revealed that baseline-enriched taxa (green) were negatively correlated with SCFA levels and clinical outcomes, whereas post-treatment taxa (red) were positively correlated.

*Phascolarctobacterium* and *Parabacteroides* were significantly negatively correlated with acetate, propionate, total SCFAs, stool frequency, and Bristol scores. *Faecalicoccus pleomorphus*, prominent in non-responders, was negatively correlated with propionate, total SCFAs, and Bristol scores.

In contrast, *Ruminococcus* sp. N15.MGS-57, *B. coprophilus*, and *Acidaminococcus* (dominant in responders post-treatment) showed strong positive correlations with SCFAs and clinical outcomes. These results suggest that SGD promotes a microbiome shift favoring beneficial taxa and metabolic profiles in responders.

### BugBase phenotype predictions

**Figure 6:**
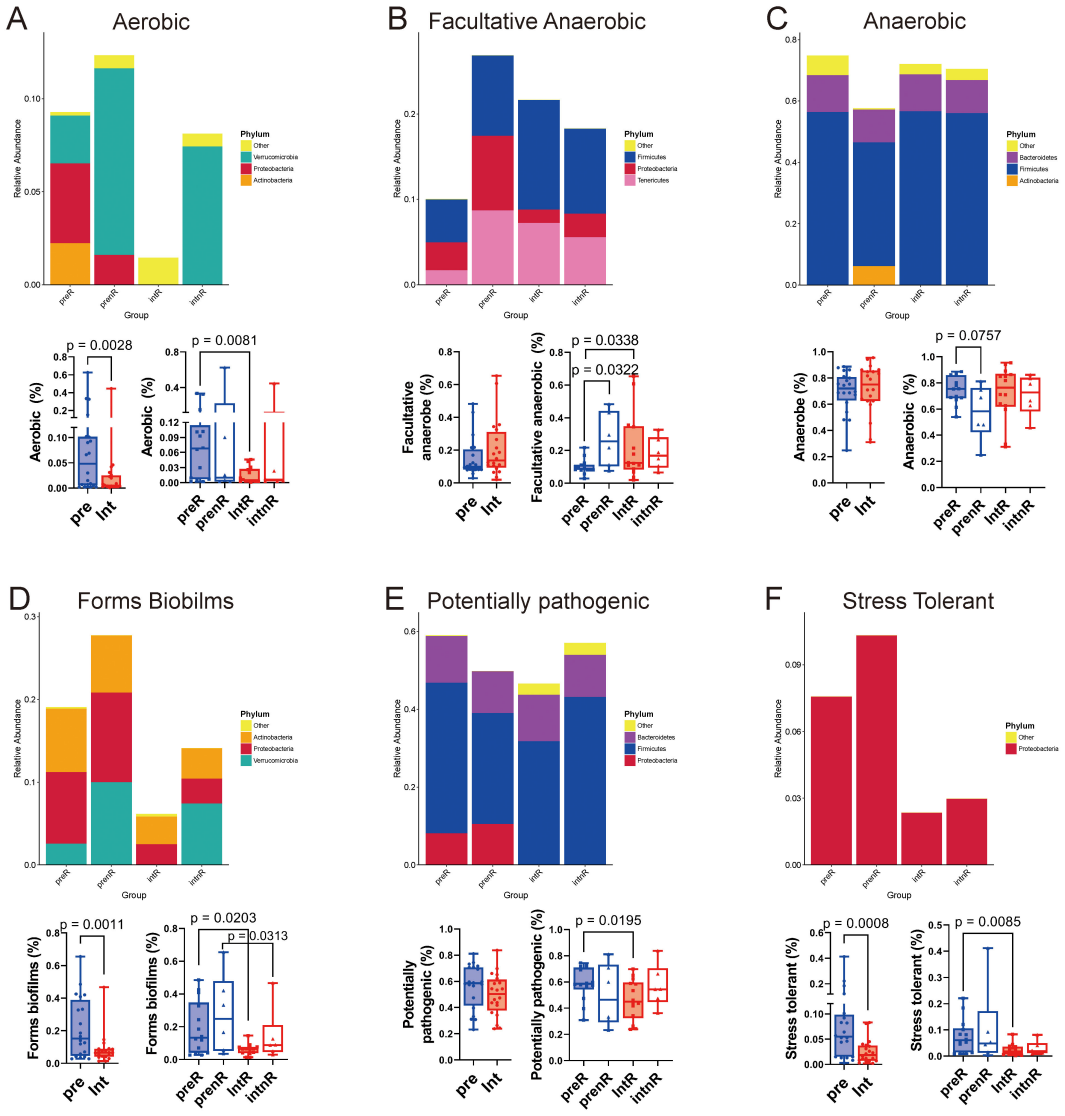
BugBase analysis of responders and non-responders before and after the SGD intervention. Relative abundance plots of the phyla involved in BugBase parameters and their corresponding proportion boxplots are shown. **(A)** aerobic bacteria; **(B)** facultative aerobic bacteria; **(C)** obligate anaerobic bacteria; **(D)** biofilm-forming bacteria; **(E)** potentially pathogenic bacteria; **(F)** oxidative stress–tolerant bacteria. Wilcoxon matched-pairs signed-rank tests were performed for pre vs. int, preR vs. intR, and prenR vs. intnR; Mann–Whitney tests for preR vs. prenR and intR vs. intnR. *p <*0.1 for plots shown.

BugBase analysis predicted reductions in aerobic, biofilm-forming, potentially pathogenic, and oxidative stress*–*tolerant bacteria after SGD intervention (**Figure 6**), particularly in responders. These reductions suggest partial restoration of a healthy anaerobic microbial environment.

At baseline, responders had elevated aerobes (**Figure 6A**) and decreased facultative anaerobes (**Figure 6B**), while non-responders showed increases in both aerobes and facultative anaerobes with reduced obligate anaerobes, indicating more severe anaerobic dysbiosis in the latter.

### Functional predictions based on KEGG

**Figure 7:**
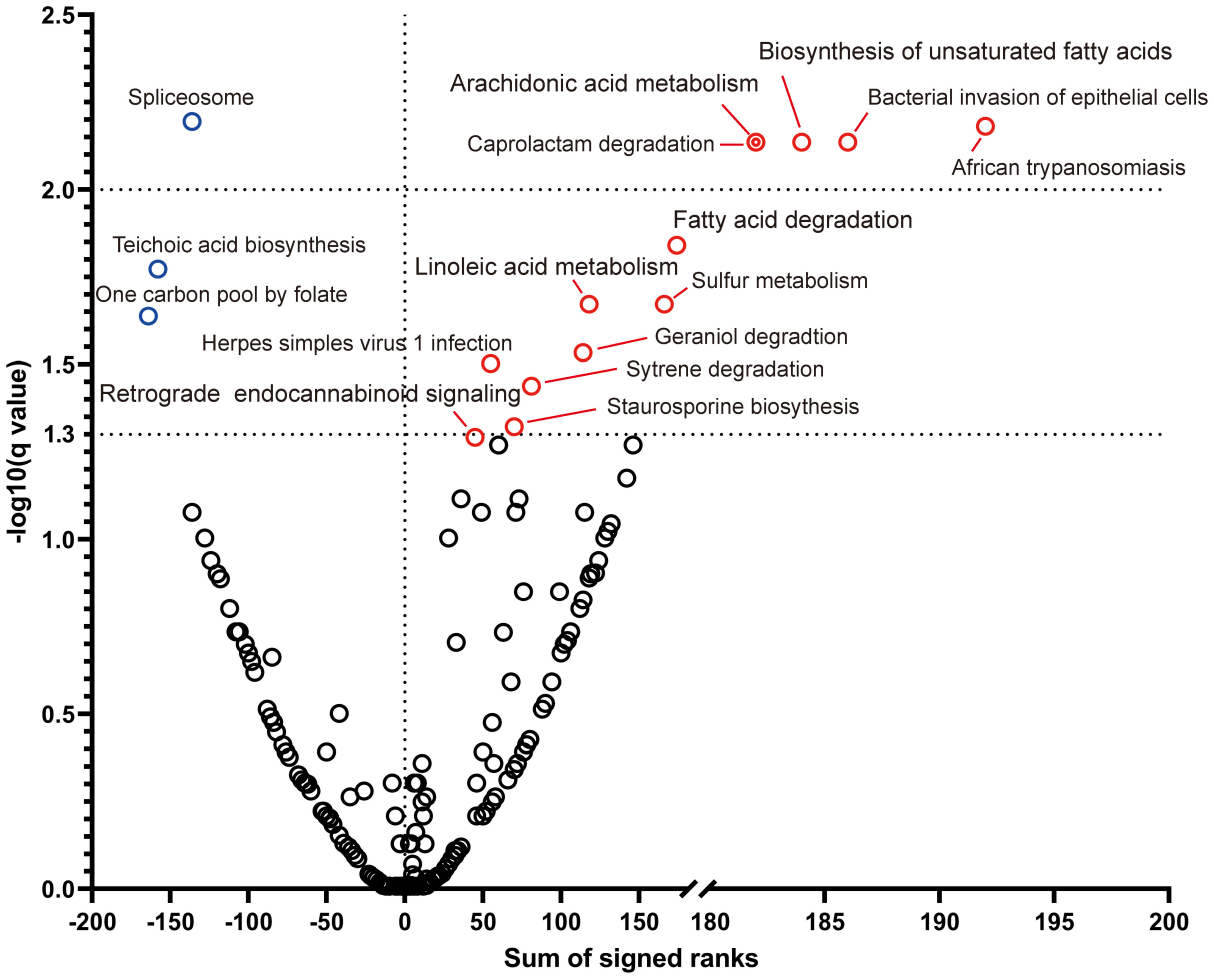
Comparison of predicted pathways before and after the SGD intervention. Volcano plot of KEGG pathways at level 3 for all patients. Wilcoxon matched-pairs signed-rank tests with FDR correction were used to calculate q-values. Notable pathway changes are labeled and colored: red indicates significant increases at baseline, and blue indicates significant increases after the SGD intervention. q <0.05 was considered significant.

PICRUSt2 functional prediction revealed downregulation of 13 KEGG level 3 pathways and upregulation of three pathways post-treatment (**Figure 7**). The downregulated pathways included bacterial invasion of epithelial cells, biosynthesis of unsaturated fatty acids, arachidonic and linoleic acid metabolism, and retrograde endocannabinoid signaling. The upregulated pathways included spliceosome function, teichoic acid biosynthesis, and the one-carbon pool by folate. These findings suggest that SGD may mitigate the epithelial barrier disruption, lipid metabolic dysregulation, and aberrant nervous system signaling associated with FC.

## Discussion

This study demonstrates that SGD intervention significantly improved stool frequency, stool consistency, and self-reported outcomes in patients with FC. These improvements were accompanied by notable shifts in fecal microbiota composition, including a reduction in *Escherichia–Shigella*, increased levels of SCFAs, and an enrichment of SCFA-producing bacteria. Additionally, SGD ameliorated lipid metabolism disturbances and reduced retrograde endocannabinoid signaling, a known inhibitor of intestinal motility. Collectively, these findings suggest that SGD exerts therapeutic effects through dual regulation of both the host and the microbiota, ultimately restoring intestinal homeostasis.

### FC and dysbiosis

At baseline, patients exhibited pronounced dysbiosis, with *Escherichia–Shigella* predominating, particularly among non-responders. This aligns with previous reports associating *Escherichia–Shigella* with FC (Yao et al., 2023) and constipation in autistic individuals (Strati et al., 2017). Higher-level taxa—including Proteobacteria, Gammaproteobacteria, Enterobacteriales, and Enterobacteriaceae—were also enriched, consistent with known signatures of dysbiosis linked to disrupted anaerobic conditions (Shin et al., 2015). Notably, *Escherichia–Shigella* remained elevated post-intervention in non-responders, who showed minimal symptomatic improvement, reinforcing its role in persistent FC (Ohkusa et al., 2019; Pan et al., 2022).

### Anti-inflammatory effects of SGD on dysbiosis

Intestinal inflammation is a key driver of dysbiosis (Lynch and Pedersen, 2016). Pro-inflammatory cytokines such as interleukin (IL)-6 and IL-1β promote metabolic shifts in epithelial cells, from mitochondrial β-oxidation to anaerobic glycolysis; this increases luminal oxygen and nitrate availability (Byndloss and Bäumler, 2018; Byndloss et al., 2017), which favors the growth of *Escherichia coli* (Winter et al., 2013; Wei et al., 2022). SGD has demonstrated anti-inflammatory effects in other models, including reduction of IL-1β and IL-6 in polycystic ovary syndrome (Chang et al., 2021). White peony root and licorice—two key components of SGD —exert immunomodulatory effects by inhibiting IL-6, nitric oxide (NO) production, and iNOS expression (He and Dai, 2011; Yang et al., 2015). Since elevated levels of IL-6 and IL-1β are also found in individuals with constipation (Wei et al., 2022; Cıralı et al., 2018), SGD’s anti-inflammatory properties likely contribute to the restoration of a balanced microbiota and the improvement of intestinal function.

### Microbial-derived endocannabinoids and neural signaling

Functional prediction analysis revealed increased retrograde endocannabinoid signaling at baseline. Endocannabinoids serve as lipid mediators that modulate excitatory neurotransmission in the gut by activating CB1 receptors (CB1Rs), thereby suppressing acetylcholine release and intestinal motility (Bisogno et al., 2003; Bashashati et al., 2015). Certain gut bacteria can produce endocannabinoid-like molecules (Degli Esposti et al., 2015); *Escherichia–Shigella* is known to generate glycerophospholipid-derived metabolites (Li et al., 2020). Moreover, *Shigella* spp. has been implicated in intractable FC via production of docosapentaenoic acid (DPA), a precursor to the endocannabinoid analog DPEA (Chen et al., 2022; Sihag and Di Marzo, 2022). The enrichment of pathways related to unsaturated fatty acid and arachidonic acid metabolism further supports the idea that there is elevated microbial endocannabinoid activity in FC. Suppression of *Escherichia–Shigella* by SGD likely reduces these metabolites, alleviating endocannabinoid-mediated disruptions in gut motility.

### A hypothetical autocrine 2-AG loop in the intestine

2-Arachidonoylglycerol (2-AG*)*, the most abundant endocannabinoid (Bashashati et al., 2015*;*Reichardt et al., 2014), can participate in an autocrine loop. In the mouse liver, 2-AG activates CB1R, inducing estrogen-related receptor gamma (ERRγ), which upregulates diacylglycerol lipase (DAGL), the enzyme responsible for 2-AG synthesis—thereby sustaining high 2-AG levels (Jung et al., 2020). Inhibition of DAGLα restores gut motility in FC models (Bashashati et al., 2015), suggesting that a similar mechanism may operate in the gut.

Given the presence of CB1Rs and DAGLα throughout the enteric nervous system (Bashashati et al., 2015) and ERRγ expression in the ileum and colon (Modica et al., 2010), we hypothesize that microbial-derived endocannabinoids trigger a 2-AG autocrine loop that sustains elevated endocannabinoid levels and inhibits gut motility. This mechanism could explain post-infectious and laxative-refractory constipation phenotypes.

### SGD-driven microbial shifts and SCFA production

SGD significantly elevated fecal SCFAs—acetate, propionate, and butyrate—all of which *are* diminished in patients with FC and increased following fecal microbiota transplantation (Mars et al., 2020*;*Pan et al., 2022*;*Su et al., 2022*;*Tian et al., 2020).

Prior to intervention, the microbiota was dominated by asaccharolytic bacteria, such as *Phascolarctobacterium*, *Parabacteroides*, *Megasphaera*, and *F. pleomorphus*, which are poorly associated with *SCFA* production (Ikeyama et al., 2020*;*Li et al., 2023*;*Reichardt et al., 2014*;*De Maesschalck et al., 2014).

Post-intervention, responders exhibited enrichment of SCFA-producing taxa, including *Bacteroides coprophilus*, *Ruminococcus* sp. *N15.MGS-57*, *Acidaminococcus*, and *Catenibacterium*—each associated with fermentation and SCFA generation (Grondin et al., 2017*;*Su et al., 2024*;*Jumas-Bilak et al., 2007*;*Kageyama and Benno, 2000).

SCFAs activate PPARγ, support epithelial mitochondrial β-oxidation, and maintain hypoxic conditions essential for gut homeostasis (Byndloss and Bäumler, 2018*;*Byndloss et al., 2017). Thus, SGD facilitated a microbial shift toward fermentative, SCFA-producing bacteria, contributing to colonic homeostasis.

### SGD-regulated bacteria interfering with the 2-AG autocrine loop

*Ruminococcus* sp. N15.MGS-57 may interfere with the 2-AG loop by modulating estrogen levels. Through β-glucuronidase activity, *Ruminococcus* increases circulating estrogen levels by deconjugating inactive estrogen forms (Zhao et al., 2022*;*Hu et al., 2023*;*Baker et al., 2017). Estrogen signaling can intersect with ERRγ pathways (Saito and Cui, 2018), potentially disrupting the ERRγ-DAGL-2AG feedback loop. SGD itself has been shown to elevate estradiol in animal models (Chang et al., 2021), further supporting this mechanism.

Additionally, *B. coprophilus*—a bile acid*–*resistant bacterium involved in primary bile acid biotransformation (Staley et al., 2017)—may regulate bile acid metabolism, thereby influencing lipid homeostasis and indirectly suppressing the 2-AG loop. Given that secondary bile acids accelerate colonic transit and inhibit *E. coli* colonization (Li et al., 2021*;*Li et al., 2018), these bacteria may synergistically restore motility via hormonal and metabolic regulation.

### Dysbiosis profiles in responders and non-responders

BugBase analysis revealed distinct dysbiosis patterns between responders and non-responders. Responders exhibited an imbalance between aerobes and facultative anaerobes, while non-responders displayed a broader dysbiosis with a marked loss of obligate anaerobes, suggesting a pan-enteric motility disorder potentially involving both the small and large intestines (Bassotti et al., 1996). Baseline LEfSe analysis identified *Escherichia–Shigella* and Clostridia- and Erysipelotrichia-related taxa as dominant in non-responders. In contrast, responders harbored aerobic and nitrate-respiring taxa such as *Pseudomonadales*, *Rhizobiales*, *Desulfovibrionales*, and *Marinobacter*, of which the latter two taxa use nitrate as an electron acceptor (Chua et al., 2018). These organisms may compete with *Escherichia–Shigella* for nitrate and oxygen, limiting their overgrowth. These findings indicate that baseline microbial composition may predict therapeutic responsiveness to SGD.

### Study limitations and future directions

A key limitation of this study is its relatively small sample size, which may reduce statistical power and limit the generalizability of the findings. Nevertheless, this work was intentionally designed as a pilot, self-controlled study to generate preliminary clinical and mechanistic evidence that could inform the design of larger randomized controlled trials. Despite the small cohort size, the consistent improvements observed in both clinical outcomes and multi-omics profiles strengthen the internal validity of our conclusions. Future investigations involving larger, multicenter cohorts—ideally incorporating randomized placebo-controlled or crossover designs—will be essential to validate these findings and to refine responder stratification based on baseline microbial signatures.

## Conclusion

This study provides compelling evidence that SGD significantly improves stool frequency and consistency in patients with FC by modulating both microbial and host physiological pathways. The findings highlight the central role of *Escherichia****–****Shigella* in FC pathogenesis and suggest that microbial-derived endocannabinoids may contribute to impaired motility by activating a host 2-AG autocrine regulatory loop. SGD interrupts this cycle through anti-inflammatory effects, microbiome restructuring, and restoration of host lipid and hormonal balance.

SGD exerts dual regulatory actions: (1) suppressing host-derived inflammatory signals and reversing dysbiosis, particularly by reducing *Escherichia****–****Shigella*; and (2) promoting SCFA-producing, bile acid**–**transforming, and estrogen-modulating bacteria that collectively inhibit the 2-AG loop and restore motility.

These findings not only support the therapeutic potential of SGD in managing FC but also illuminate broader host–microbe interactions in gut motility regulation. They open avenues for integrating traditional medicine with microbiome-targeted strategies to develop more personalized and effective treatments for FC—especially among women, who are disproportionately affected.

## Data Availability

The data that support the findings of this study are openly available in the National Microbiology Data Center at https://nmdc.cn/resource/genomics/sra/detail/NMDC10018777, reference number (NMDC10018777).
